# Supplementary material for: The role of heat shock proteins in HIV-1 pathogenesis: a systematic review investigating HSPs-HIV-1 correlations and interactions
Source: PeerJ. 2024 Sep 19;12:e18002. doi: 10.7717/peerj.18002 (PMC11416755; doi:10.7717/peerj.18002)
Supplement: Supplemental Information 2 [file peerj-12-18002-s002.docx]

**The rationale for conducting the systematic review / meta-analysis:**

The human immunodeficiency virus (HIV) type 1 (HIV-1) pandemic is a significant public health emergency since the associated immunodeficiency allows a myriad of infectious diseases that may spread within and between different countries and geographic regions (Esbjörnsson et al., 2019; Waymack & Sundareshan, 2023). Around 40.4 million individuals have died from HIV, and 85.6 million have been infected ever since the beginning of the pandemic (World Health Organization, 2023). The management of HIV-1 infection mainly relies on antiretroviral therapy (ART), which typically includes three medications from two ARV drug classes (Tyler R. Kemnic & Peter G. Gulick, 2023). However, despite the availability of ART, the management of HIV-1 infection currently faces several challenges, including the emergence of drug resistant strain which might reduce the effectiveness of various ARV drug classes (Khairunisa et al., 2020; World Health Organization, 2021; Bertagnolio et al., 2022); therefore, exploring novel therapeutic strategies for the management of HIV-1 infection is urgent.

Heat shock proteins (HSPs) are ubiquitous and conserved family of proteins which maintains cell proteostasis and protects cells from stresses (Iyer et al., 2021; Hu et al., 2022; Zhang & Yu, 2022). In viral infection, HSPs have been suggested as one of the critical actors in antigen presentation and antiviral innate immunity response and have been implicated in several viral replication processes, including dengue, influenza, and hepatitis C virus (Ujino et al., 2009; Manzoor et al., 2014; Taguwa et al., 2015; Wan et al., 2020a).

Recent studies have hinted at a significant connection between HSPs and HIV-1 infection pathogenesis (Iyer et al., 2021; Zhang & Yu, 2022). Given the significance of HSPs in cellular processes yet their enigmatic role in the framework of HIV-1 infection, a comprehensive review of their interplay is essential. Herein, we aim to delve into the current literature to clarify the role of HSPs in HIV-1 pathogenesis, offering insights that might pave the way to a deeper understanding of HIV-1-host interactions and possible novel therapeutic strategies.

**The contribution that it makes to knowledge in light of previously published related reports, including other meta-analyses and systematic reviews:**

In this review, we underlined the involvement of several HSPs as critical molecular chaperone in different stages of HIV-1 infection, particularly the HSP40, HSP70, HSPBP1, and HSP90. **No previous systematic review has summarized the interaction between HSPs and HIV-1 infecetion**. However, all studies included in this review were conducted in vitro, and the clinical implications remain to be determined. We hope that in vivo studies to validate the clinical significance of these interactions and their potential as targets for therapeutic intervention are within reach.
